# Transcriptome Analysis Revealed a Highly Connected Gene Module Associated With Cirrhosis to Hepatocellular Carcinoma Development

**DOI:** 10.3389/fgene.2019.00305

**Published:** 2019-04-02

**Authors:** Shan Shan, Wei Chen, Ji-dong Jia

**Affiliations:** ^1^Liver Research Center, Beijing Friendship Hospital, Capital Medical University, Beijing, China; ^2^Beijing Key Laboratory of Translational Medicine on Liver Cirrhosis, Beijing Friendship Hospital, Capital Medical University, Beijing, China; ^3^National Clinical Research Center for Digestive Disease, Beijing Friendship Hospital, Capital Medical University, Beijing, China; ^4^Experimental and Translational Research Center, Beijing Friendship Hospital, Capital Medical University, Beijing, China; ^5^Beijing Key Laboratory of Tolerance Induction and Organ Protection in Transplantation, Beijing Friendship Hospital, Capital Medical University, Beijing, China

**Keywords:** cirrhosis, hepatocellular carcinoma, transcriptome, module, prognosis

## Abstract

**Introduction:**

Cirrhosis is one of the most important risk factors for development of hepatocellular carcinoma (HCC). Recent studies have shown that removal or well control of the underlying cause could reduce but not eliminate the risk of HCC. Therefore, it is important to elucidate the molecular mechanisms that drive the progression of cirrhosis to HCC.

**Materials and Methods:**

Microarray datasets incorporating cirrhosis and HCC subjects were identified from the Gene Expression Omnibus (GEO) database. Differentially expressed genes (DEGs) were determined by GEO2R software. Functional enrichment analysis was performed by the clusterProfiler package in R. Liver carcinogenesis-related networks and modules were established using STRING database and MCODE plug-in, respectively, which were visualized with Cytoscape software. The ability of modular gene signatures to discriminate cirrhosis from HCC was assessed by hierarchical clustering, principal component analysis (PCA), and receiver operating characteristic (ROC) curve. Association of top modular genes and HCC grades or prognosis was analyzed with the UALCAN web-tool. Protein expression and distribution of top modular genes were analyzed using the Human Protein Atlas database.

**Results:**

Four microarray datasets were retrieved from GEO database. Compared with cirrhotic livers, 125 upregulated and 252 downregulated genes in HCC tissues were found. These DEGs constituted a liver carcinogenesis-related network with 272 nodes and 2954 edges, with 65 nodes being highly connected and formed a liver carcinogenesis-related module. The modular genes were significantly involved in several KEGG pathways, such as “cell cycle,” “DNA replication,” “p53 signaling pathway,” “mismatch repair,” “base excision repair,” etc. These identified modular gene signatures could robustly discriminate cirrhosis from HCC in the validation dataset. In contrast, the expression pattern of the modular genes was consistent between cirrhotic and normal livers. The top modular genes TOP2A, CDC20, PRC1, CCNB2, and NUSAP1 were associated with HCC onset, progression, and prognosis, and exhibited higher expression in HCC compared with normal livers in the HPA database.

**Conclusion:**

Our study revealed a highly connected module associated with liver carcinogenesis on a cirrhotic background, which may provide deeper understanding of the genetic alterations involved in the transition from cirrhosis to HCC, and offer valuable variables for screening and surveillance of HCC in high-risk patients with cirrhosis.

## Introduction

Hepatocellular carcinoma accounts for 90% of all primary liver malignancies (Mittal and El-Serag, 2013; Galle et al., 2018), posing a serious threat to human health and quality of life. Worldwide, most patients with HCC have underlying cirrhosis of various etiologies (Fattovich et al., 2004; Beste et al., 2015; Walker et al., 2016). Growing clinical evidence shows that removal or control of the injurious factors, such as hepatitis B or C virus, can reduce but not eliminate the risk of HCC (Casado et al., 2013; Marcellin et al., 2013; Xu et al., 2015; Sun et al., 2017). Therefore, it is important to understand the molecular mechanisms that drive the progression of cirrhosis to HCC.

Hepatocellular carcinoma occurs as a consequence of the complex interplay between multiple genetic determinants (Sanyal et al., 2010). Previous studies have found that aberrations in genetic molecules pertaining to oxidative stress, EMT, inflammatory response, cellular senescence, or telomere dysfunction may contribute to the progression of cirrhosis to HCC (Ramakrishna et al., 2013). In addition, the Wnt/β-catenin, p53, pRb, MAPK, RAS, and JAK/STAT pathways are also reported to be canonical molecular pathways in HCC development (Aravalli et al., 2008). However, different studies often yield diverse results and the global view on the landscape of genomic changes is still not very clear.

With the aid of high-throughput detection techniques, all expressed genetic molecules in a given liver tissue sample can be simultaneously detected over a wide quantitative range (Mas et al., 2009; Villanueva et al., 2011; Yildiz et al., 2013; Wang et al., 2014; Lee, 2015; Schulze et al., 2015; Villanueva et al., 2015; Diaz et al., 2018; Shen et al., 2018). High-throughput sequencing and microarray technologies allow investigators to simultaneously measure the changes of genome-wide genes under certain biological conditions. These approaches usually generate large “interesting” gene lists. By using biological knowledge accumulated in public databases (e.g., KEGG^1^), it is possible to systematically dissect large gene lists in an attempt to assemble a summary of the most enriched and pertinent biology. Therefore, integrated analyses of multiple datasets generated from different studies may help us to identify reliable and reproducible genetic alterations involved in the development of HCC on a cirrhotic background.

Therefore, our present study used multiple bioinformatics tools to systematically integrate publicly available transcriptomic datasets and performed high-throughput gene expression comparisons between HCC and benign cirrhotic tissues.

## Materials and Methods

### Retrieval of Microarray Datasets on Cirrhosis and HCC From Public Database

First, we searched and retrieved transcriptome profiles of cirrhotic and HCC tissues from GEO which is a public functional genomics data repository, allowing users to query, locate, review, and download studies and gene expression profiles of interest (Barrett et al., 2013).

**FIGURE 1:**
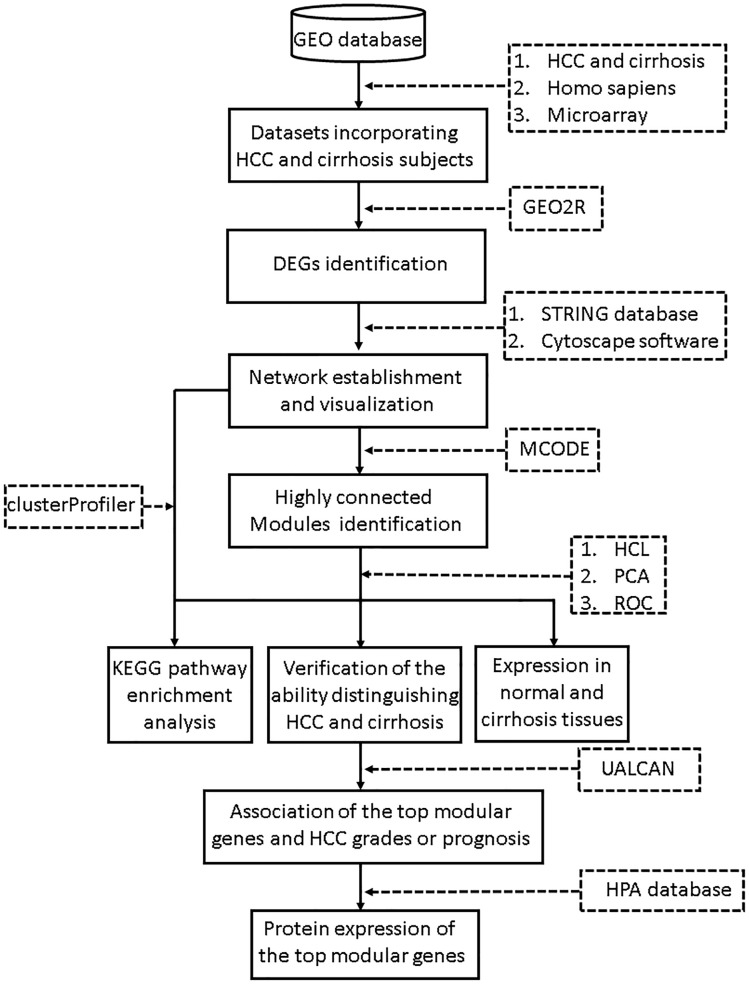
Workflow for bioinformatics analysis. GEO, Gene Expression Omnibus; HCC, hepatocellular carcinoma; STRING, Search Tool for the Retrieval of Interacting Genes; MCODE, Molecular Complex Detection; HCL, hierarchical clustering; PCA, principal component analysis; KEGG, Kyoto Encyclopedia of Genes and Genomes; HPA, Human Protein Atlas. ROC, receiver operating characteristic.

The search terms we used included “cirrhosis” and “HCC.” Studies were considered eligible for analysis if: (1) studies contained both cirrhosis and HCC tissues; (2) species was limited to Homo sapiens; and (3) platform was limited to microarray. Then the retrieved datasets were further screened by manual retrieval. Our workflow for bioinformatics analysis of publicly available datasets is illustrated in Figure 1.

### Identification of DEGs Related to Liver Carcinogenesis From the Retrieved Microarray Datasets

Differentially expressed genes between cirrhosis and HCC tissues were defined as liver carcinogenesis-related genes that may have important implications in driving cirrhosis to HCC.

Gene expression in all the datasets was normalized by the antilog-transformed RMA algorithm. GEO query and limma R packages contained in GEO2R, which allows gene expression analysis of published microarray datasets, was used to determine the DEGs between cirrhosis and HCC tissues (Davis and Meltzer, 2007). FDR < 0.05 and FC > 1.5 were considered as the cutoff values for DEG screening. The overlapping DEGs in datasets were retained for further analyses.

### Functional Specification of the Identified DEGs Related to Liver Carcinogenesis

To identify and visualize enriched KEGG pathways for the candidate gene sets, clusterProfiler, which is an R package for comparing biological themes among gene clusters, was employed (Yu et al., 2012). Fisher’s exact test followed by the Benjamini correction was performed and an adjusted *P*-value of <0.05 was set as the cutoff criterion.

### Establishment of the LiverCarcinogenesis-Related Network andIts Modules

The internal regulatory relationships between the identified liver carcinogenesis-related genes were predicted by the STRING database (confidence score > 0.4) (Szklarczyk et al., 2017). Liver carcinogenesis-related network was established and visualized with Cytoscape software (Shannon et al., 2003).

We used the MCODE plug-in in the Cytoscape software (Bader and Hogue, 2003) to screen the modules concealed in the liver carcinogenesis-related network with the following criteria: Max. depth = 100, K-Core = 2, mode score cutoff = 0.2, and degree cutoff = 2. Likewise, the functional specification of the identified module was determined with the clusterProfiler package as mentioned above. An adjusted *P*-value of <0.05 was considered statistically significant.

### Verification of the Identified Modules for Discriminating Cirrhosis From HCC

We used three of datasets (GSE89377, GSE17548, and GSE98383) to mine modules from the liver carcinogenesis-related network; and used the remaining dataset (GSE56140) to validate the findings. To verify the ability of the identified modules to discriminate cirrhosis from HCC subjects, we performed hierarchical clustering analysis by using R with the complete linkage method and the expression of modular genes as a distance metric. To verify the results of hierarchical clustering, we applied the identified modular genes that were considered as observed variables to PCA plots. PCA was conducted with the ggbiplot package in R. The first two principal components (PCs) were then subjected to binary logistic regression analysis to calculate the predicted probability which was applied to the receiver operating characteristic (ROC) curve analysis implemented by SPSS 20 (IBM, United States). Area under curve (AUC) was calculated to determine the predictive performance of the identified gene module. In order to reduce sampling bias, the modules were screened from any three out of the four datasets and repeated evaluations of their discriminant ability were performed using the remaining dataset.

### Comparison of the Identified Modular Genes in Normal and Cirrhotic Samples

We used GEO2R software to determine the expression differences between any two groups in dataset. An FDR of <0.05 and an FC of >1.5 were considered as the cutoff values for DEG screening. Modular gene expression in normal, cirrhotic, and HCC samples were visualized by using a heatmap drawn with MeV software^2^.

### Analyses of the Association Between the Top Modular Genes and HCC Histological Grade or Clinical Outcome

Modular genes with FC > 3 between cirrhosis and HCC tissues in all GEO datasets were considered as the top modular genes. Association of the top modular genes and HCC grades or prognosis was analyzed by using UALCAN (Chandrashekar et al., 2017), which is an interactive web-portal for exploring the association between tumor subgroup gene expression and survival in TCGA^3^. Expression differences of top modular genes between normal and different tumor grades were analyzed using the statistical method built-in the UALCAN web-software; a *P*-value of <0.05 was considered significant.

According to the TPM expression values, the top modular genes were divided into a high expression group (with TPM values above upper quartile) and a low expression group (with TPM values below lower quartile). With information on the association between the gene expression and survival profiles documented in TCGA, Kaplan–Meier survival analyses were performed and overall survival plots were generated. The difference between high gene expression and low gene expression was compared by log-rank test; a *P*-value of < 0.05 was considered significant.

### *In silico* Analysis of the Top Modular Members in Normal and HCC Specimens

Protein expression and distribution of the top modular genes in human liver tissue were searched in the HPA ^4^ database (Uhlen et al., 2015).

## Results

### Retrieved Microarray Datasets Pertaining to Cirrhosis and HCC

According to the retrieval criteria, four microarray datasets (Table 1) containing a total of 95 benign (cirrhosis) and 98 malignant (HCC) subjects were found from the GEO database. GSE98983 dataset was produced by Affymetrix Human Genome U133 Plus 2.0 Array (GPL570); GSE89377 by Illumina HumanHT-12 V3.0 expression beadchip (GPL6947); GSE17548 by Affymetrix Human Genome U133 Plus 2.0 Array (GPL570); and.GSE56140 by Illumina HumanHT-12 V3.0 expression beadchip.

**Table 1:** Detailed information of the four microarray datasets used in the present study.

ID	Platform	Sample	Etiology	References
GSE98383	GPL570	LC = 29; HCC = 11	HDV	Diaz et al., 2018
GSE89377	GPL6947	LC = 12; HCC = 35	HBV, HCV, alcohol, others	Shen et al., 2018
GSE17548	GPL570	LC = 20; HCC = 17	HBV, HCV, HBV+HDV, others	Yildiz et al., 2013
GSE56140	GPL18461	LC = 34; HCC = 35	HBV, HCV	Villanueva et al., 2011

*LC, liver cirrhosis; HCC, hepatocellular carcinoma; HBV, hepatic B virus; HCV, hepatic C virus; HDV, hepatic D virus.*

### DEGs Related to Liver Carcinogenesis

**FIGURE 2:**
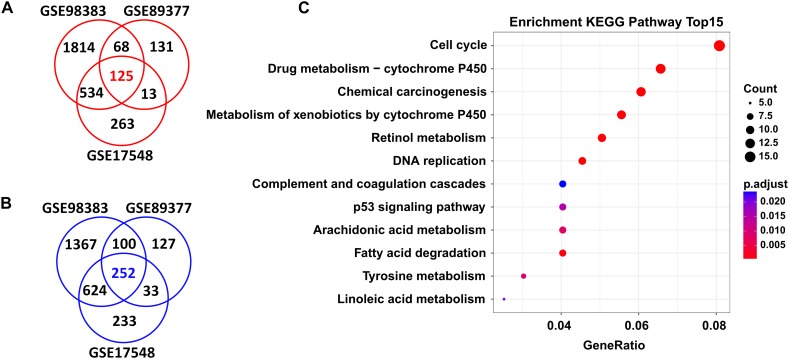
DEGs shared in three independent microarray datasets and their potential functions. A total of 125 upregulated genes **(A)** and 252 downregulated genes **(B)** were shared between the GSE98383, GSE89377, and GSE17548 datasets when comparing HCC to cirrhosis subjects. Red indicates upregulation and blue represents downregulation. DEGs were determined using GEO2R software. FDR < 0.05 and FC > 1.5 were considered as the cutoff values. **(C)** Significantly enriched KEGG pathways of the total shared DEGs. ClusterProfiler package was selected to perform KEGG pathway enrichment analysis. Adjusted *P*-values of <0.05 were considered statistically significant.

In total, we found 125 upregulated and 252 downregulated genes (adjusted *P* < 0.05 and FC > 1.5) in HCC tissues when compared with cirrhosis tissues. These DEGs were shared by the three independent datasets (GSE98383, GSE89377, and GSE17548) (Figure 2A,B and Supplementary Table S1).

### Functional Specification of DEGs Related to Liver Carcinogenesis

Functional enrichment analysis revealed that these DEGs were significantly enriched in several KEGG pathways; as shown in Figure 2C, the top ones were “cell cycle,” “drug metabolism-cytochrome P450,” “chemical carcinogenesis,” “metabolism of xenobiotics by cytochrome P450,” “retional metabolism,” “DNA replication,” “complement and coagulation cascades,” “p53 signaling pathway,” etc. Detailed information of these pathways is listed in Supplementary Table S2.

### Network-Based Modules Involved in Liver Carcinogenesis

**FIGURE 3:**
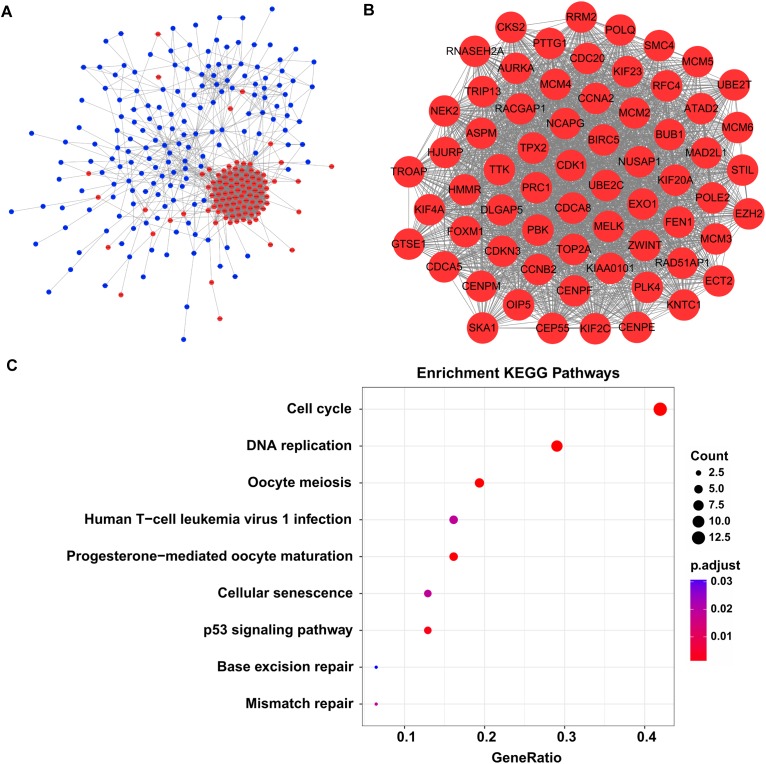
The liver carcinogenesis-related module and its biological functions. **(A)** Liver carcinogenesis-related network. The internal interactions between DEG pairs were mined using STRING database and the network was visualized using Cytoscape software. Red nodes signify upregulated genes and blue nodes indicate downregulated genes. The edges between any two nodes represent internal interactions. **(B)** Liver carcinogenesis-related module. Members in this module are highly connected. All the modular genes were upregulated in HCC tissues. **(C)** Significantly enriched KEGG pathways of the modular genes. ClusterProfiler package was selected to perform functional specification. Adjusted *P*-values of <0.05 were considered statistically significant.

By screen against the STRING database, totally 2954 interactions were found among 272 DEGs, which was visualized by using Cytoscape software. The network layout was arranged with the Allegro Spring-Electric plug-in in Cytoscape software. As shown in Figure 3A, the upregulated genes in the liver carcinogenesis-related network were highly connected, suggesting a core role in the whole network.

Then we used the MCODE plug-in to mine the highly clustered modules from this network. As expected, a module holding a higher connectivity (cluster score = 64.1) was identified, with 65 nodes and 1955 edges. Interestingly, all the modular genes were notably upregulated in HCC tissues compared with cirrhotic tissues, signifying their roles in liver carcinogenesis (Figure 3B and Supplementary Table S3). These modular genes were involved in several KEGG pathways including “cell cycle,” “DNA replication,” “oocyte meiosis,” “human T-cell leukemia virus 1 infection,” “progesterone-mediated oocyte maturation,” “cellular senescence,” “p53 signaling pathway,” “base excision repair,” and “mismatch repair” (Figure 3C and Supplementary Table S4).

### Modular Gene Signatures Discriminating Cirrhosis From HCC

**FIGURE 4:**
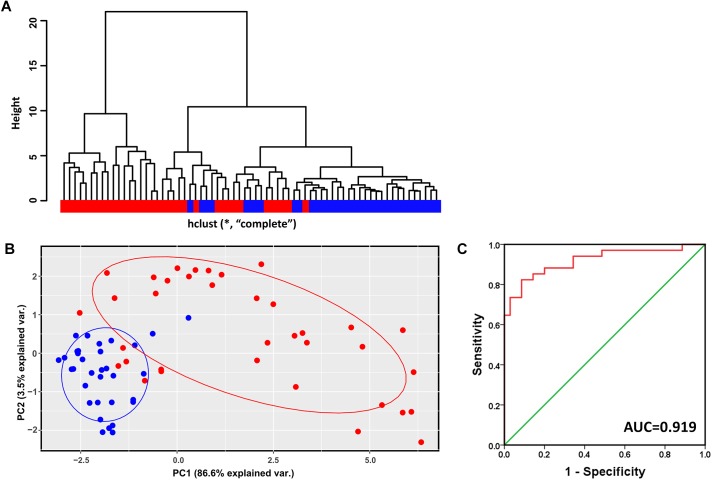
Verification of the identified modules for discriminating cirrhosis from HCC. **(A)** Hierarchical clustering dendrogram of all the subjects in GSE56140 (distance metric: modular gene expressions, linkage method: complete). Red represents HCC subjects and blue represents cirrhosis subjects. **(B)** PCA plot of all the subjects in GSE56140. HCC subjects are labeled in red and cirrhosis subjects are labeled in blue. The ellipse shows 95% confidence intervals. **(C)** The first two PCs were subjected to binary logistic regression analysis to calculate the predicted probability which was applied to the ROC analysis. AUC, area under curve.

Hierarchical clustering analysis of the validation dataset (GSE56140, 34 cirrhosis subjects and 35 HCC subjects) showed that subjects with the same diagnosis were inclined to be evidently more clustered (Figure 4A). In agreement with the result of hierarchical clustering, as shown in Figure 4B, the PCA plot clearly distinguished cirrhosis from HCC with a small overlap. The first two PCs that were the most informative, accounting for approximately 86.6 and 3.5% of the total observed variances, respectively. ROC analysis revealed an AUC of 0.919, indicating the identified modular genes, to some extent, could be a combined predictor of cirrhosis to HCC development (Figure 4C).

We next used any three out of the four datasets as training datasets to mine liver carcinogenesis-related modules. As shown in Supplementary Figures S1–S3, all the identified modules, as the module screened from GSE98383, GSE89377, and GSE17548, were characterized by upregulated genes and high connectivity; members in these modules identified from different training datasets were largely overlapped. In addition, hierarchical clustering, PCA and ROC analyses, to a large extent, could distinguish cirrhotic subjects from HCC subjects (Supplementary Figures S4–S6). These results indicated that the identified liver carcinogenesis-related module was not by chance and the ability of discriminating cirrhosis from HCC was relatively robust.

### Characterization of the Modular Gene Expression Patterns in Normal, Cirrhotic, and HCC Livers

**FIGURE 5:**
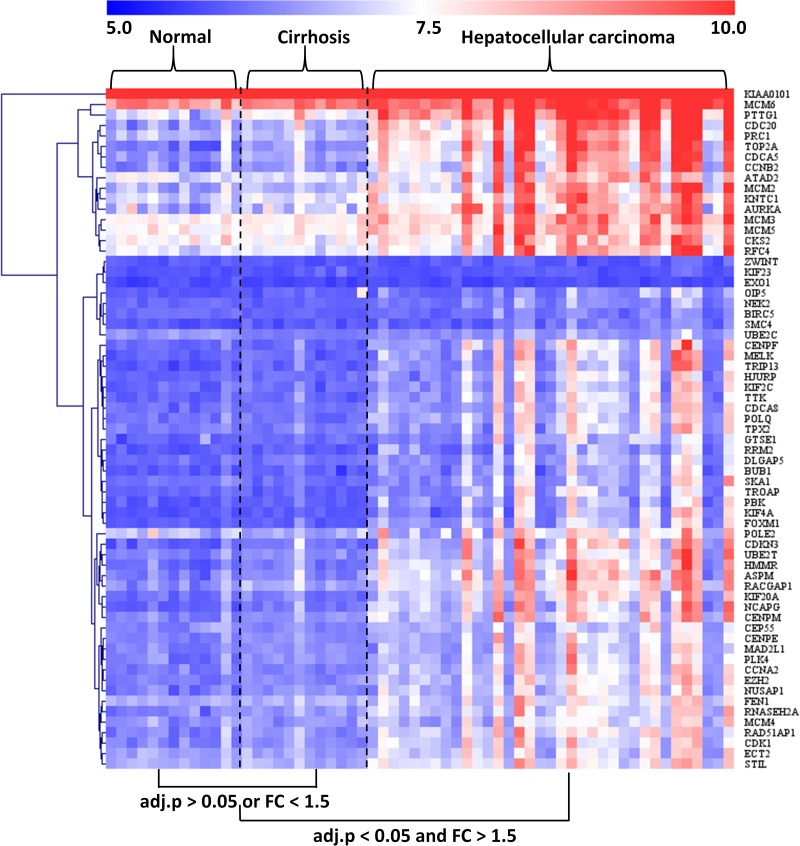
Heatmap of modular gene expression in normal, cirrhotic, and HCC livers. GSE89377 dataset contained 13, 12, and 35 normal, cirrhosis, and HCC subjects, respectively. Modular gene expression in GSE89377 was normalized by antilog-transformed RMA value. Heatmap of modular gene expression was generated by MeV software. Differential gene expression analysis between the two groups was performed using GEO2R. FC represents fold-change. Red indicates high expression and blue signifies low expression.

The GSE89377 dataset contained not only cirrhosis (*n* = 12) and HCC (*n* = 35) subjects but also normal (*n* = 13) subjects. Therefore, we used this dataset to analyze the modular gene expression pattern in normal, cirrhotic, and HCC livers. As shown in Figure 5, while the modular genes being differentially expressed between HCC and cirrhosis/normal livers (adjusted *P* < 0.05 and FC > 1.5), there were no significant differences in expression between cirrhosis and normal livers (adjusted *P* > 0.05 or FC < 1.5).

### Association of Top Modular Gene Signatures With HCC Onset, Progression, and Prognosis

We next focused on the top modular gene signatures because their expression was highly dysregulated in the HCC tissues of all four datasets considered in this study. In total, five modular genes including TOP2A, CDC20, PRC1, CCNB2, and NUSAP1 satisfied the criterion stated in Section “Materials and Methods,” therefore they were considered as the top modular gene signatures.

**FIGURE 6:**
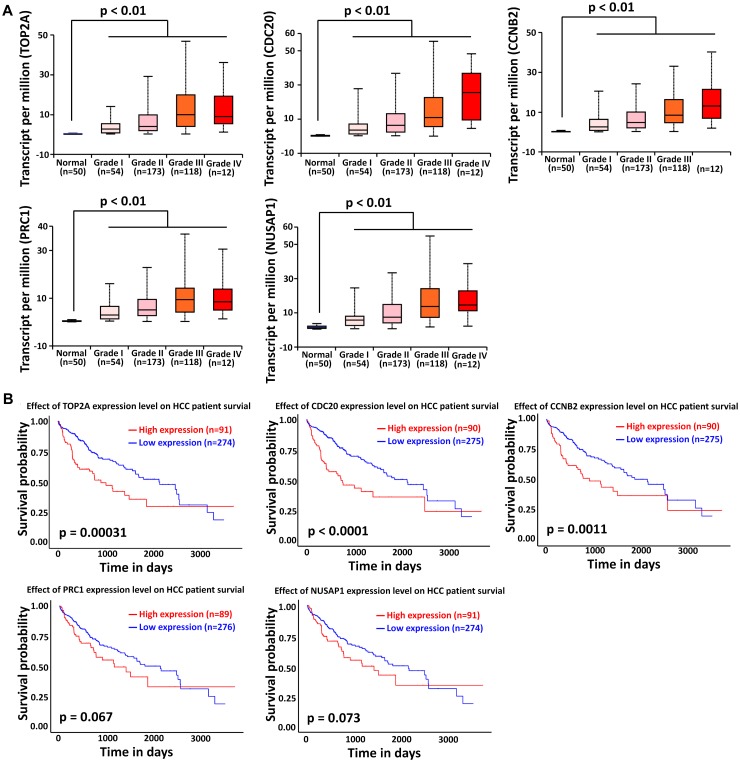
Association of TOP2A, CDC20, PRC1, CCNB2, and NUSAP1 expression with HCC progression and prognosis. **(A)** Validation of the association between the expression levels of TOP2A, CDC20, PRC1, CCNB2, and NUSAP1 and the pathological stages of HCC (based on TCGA data in UALCAN web-tool). **(B)** Kaplan–Meier analysis of overall survival in HCC patients in TCGA liver cancer dataset based on TOP2A, CDC20, PRC1, CCNB2, and NUSAP1 expression.

The liver cancer datasets in TCGA database contained 50 normal subjects, 54 HCC subjects with grade I, 173 HCC subjects with grade II, 118 HCC subjects with grade III, and 12 HCC subjects with grade IV. As shown in Figure 6A, all the top modular gene signatures were significantly upregulated in each HCC grade group, compared with the normal group (*P* < 0.05), and the expression of all genes increased in a stepwise manner with the HCC progress, suggesting a close relationship between the five genes and HCC onset and progression.

To investigate if the top modular gene signatures affected overall survival in patients with HCC, we performed a survival prediction by Kaplan–Meier curve analysis embedded in UALCAN web-tool. As shown in Figure 6B, high expression of TOP2A, CDC20, and CCNB2 protein was significantly associated with poor survival time in HCC patients (*P* < 0.01). Although lower expression of PRC1 and NUSAP1 tended to be associated with better outcome in HCC patients, significant differences were not achieved (*P* > 0.05).

### Protein Expression and Distribution of TOP2A, CDC20, PRC1, CCNB2, and NUSAP1 in HCC Livers

In the HPA database, we were able to find normal and HCC sections from several patients with staining for the top modular proteins. Antibodies used in the HPA database were: TOP2A (HPA006458), CDC20 (CAB004525), PRC1 (HPA034521), CCNB2 (CAB009575), and NUSAP1 (HPA043904).

**FIGURE 7:**
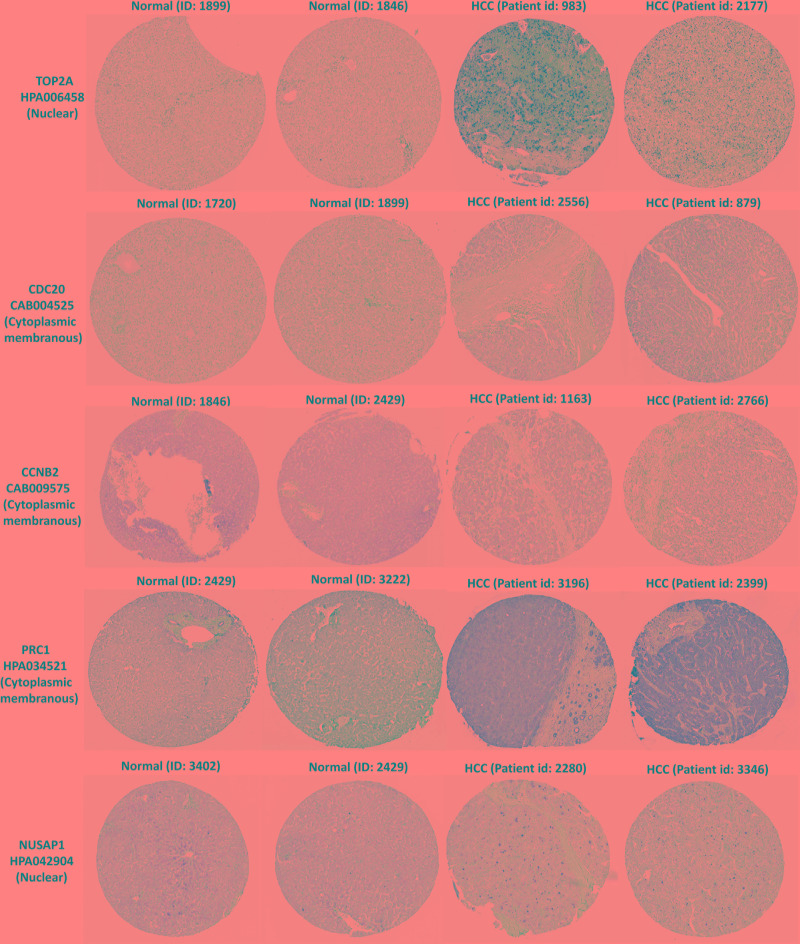
TOP2A, CDC20, PRC1, CCNB2, and NUSAP1 proteins expression in normal and HCC tissues from the HPA database. Selected immunohistochemistry images of proteins (TOP2A, PRC1, and NUSAP1) detected in HPA database that showed almost negative staining in normal tissue but rather high expression in HCC tissue. Magnification, 100×. HCC, hepatocellular carcinoma; HPA, Human Protein Atlas.

Immunohistochemistry for the five members in the HPA database showed that TOP2A and NUSAP1 highly expressed in HCC cell nuclei but almost undetectable in normal tissue, whereas PRC1 highly expressed in HCC cytoplasm and plasma membrane but undetectable in normal liver tissue. Although CDC20 and CCNB2 exhibited higher rate of expression in HCC cytoplasm and plasma membrane, their abundance was very low (Figure 7). Hence, TOP2A, PRC1, and NUSAP1 have the potential to be liver biopsy-based markers for screening HCC high-risk patients with cirrhosis.

## Discussion

Our current study systematically integrated four independent microarray datasets that contains cirrhosis and HCC tissues. By performing a series of bioinformatics analyses, we found a highly connected module covering 65 HCC risky genes, which could robustly distinguish cirrhosis from HCC; the top modular genes were highly associated with HCC onset and development and prognosis.

The module identified in the present study was highly connected; by functional enrichment analysis, the modular genes were found to be involved in several KEGG pathways. Mismatch repair pathway usually corrects insertion/deletion loops and base–base mismatches generated during DNA replication and recombination; base excision repair pathway is the main repair mechanism for DNA damage; the two pathways together with cell cycle and DNA replication pathways are the foundational mechanisms determining cell fate associated with carcinogenesis (Macdonald et al., 1998; Bisteau et al., 2014; Mjelle et al., 2015; Liu Z. et al., 2018). P53 signaling pathway inhibition has been widely reported to be required for liver cancer initiation (Cao et al., 2018; Dhar et al., 2018). Cellular senescence, a process of cell proliferation arrest in response to multifarious stimuli, which can modify the microenvironment of tissues, has been reported to be closely associated with cancer onset of multiple tissues including liver (Kim and Park, 2019). Since the increase of senescent cells, it is likely that the preneoplastic setting of the cirrhotic background may provide a conducive environment for cellular transformation, which should be further investigated. Although human T-cell leukemia virus 1 infection pathway has not been reported to be linked to HCC, but the upregulation of modular genes involved in this pathway such as CDC20, MAD2L1, and PTTG1 have been confirmed to promote HCC development and progression (Cho-Rok et al., 2006; Li et al., 2014; Li Y. et al., 2017). Except the pathways mentioned above, “oocyte meiosis” and “progesterone-mediated oocyte maturation” are two main KEGG pathways which the identified modular gene were enriched in. Oocyte meiosis and progesterone-mediated oocyte maturation pathways have also been found to be associated with HCC by integrated analysis of microarray studies (Li L. et al., 2017; Wang F. et al., 2017). However, the causal association between oocyte meiosis and HCC onset should be investigated in further study.

The top modular genes, TOP2A, CDC20, PRC1, CCNB2, and NUSAP1, were highly associated with HCC onset and development; high expression of TOP2A, CDC20, or CCNB2 was correlated with poor survival time in TCGA liver cancer patients, implying their potential as biopsy-based prognostic markers. Consistent with our findings in the HPA database, previous studies have also found that TOP2A (Panvichian et al., 2015; Ang et al., 2016; Xu et al., 2016; Li et al., 2018; Wang et al., 2018; Zhou et al., 2018), CCNB2 (Liu S. et al., 2018; Zhou et al., 2018), CDC20 (Li et al., 2014; Jin et al., 2015; Li L. et al., 2017; Yan et al., 2017; Fan et al., 2018), PRC1 (Chen et al., 2016; Wang Y. et al., 2017; Liu X. et al., 2018), and NUSAP1 (Zhang et al., 2013; Roy et al., 2018; Zhou et al., 2018) are overexpressed in HCC but are almost undetectable in non-tumorous livers. TOP2A has been previously confirmed to correlate with advance histological grading, microvascular invasion, early age onset, shorter patient survival, and chemoresistance of HCC (Wong et al., 2009). High expression of CDC20 in HCC patients is associated with shorter overall survival (Fan et al., 2018); silencing of CDC20 expression significantly inhibits HCC cell proliferation and tumor growth (Li et al., 2014, Liu et al., 2015). PRC1 in HCC tissue is regulated by Wnt3a signaling, exerting an oncogenic effect by promoting cancer proliferation, stemness, metastasis, and tumorigenesis; high expression of PRC1 was associated with early HCC recurrence and poor patient outcome (Chen et al., 2016; Wang Y. et al., 2017). Finally, NUSAP1 expression in the surgical margins of HCC is closely correlated to early postoperative recurrence and could serve as an indicator for predicting early recurrence of HCC (Zhang et al., 2013).

Despite studies devoted to decoding the process of cirrhosis development to HCC have been extensively reported, integrated studies based on multiple datasets are rare. Prior to this current work, only one study had been reported by Jiang et al. (2017) who performed a weighted gene co-expression network analysis based on five independent gene expression profiles and identified six modules contributing to HCC progression. They found hub genes in the identified modules were mainly cytokines, such as chemokine (C-C motif) ligand 22 and interleukin-19 (Jiang et al., 2017). However, our study found only one highly connected module that closely involved in the canonical carcinogenesis-associated pathways. The following reasons may at least partly explain such difference. First, cirrhotic tissues in each microarray dataset included in our study were benign tissues only and not mixture of benign and para-carcinoma tissues. Second, the microarray profiles in our study were generated by more than one platform and etiologies covered HBV, HCV, HDV, alcohol, and others. In order to obtain more reliable results, gene expression datasets from different microarray platforms and etiologies were considered; the overlapped DEGs were retained for further analysis; datasets were separated into training and test datasets, and the ability of the identified functional module distinguishing cirrhosis from HCC was validated in the test dataset; resampling and repeated evaluations obtained robust results; moreover, the associations between the expression of the top modular genes and HCC progression and prognosis were determined in other liver cancer datasets.

## Conclusion

Our present study systematically integrated multiple microarray gene expression profiles and found a module associated with liver carcinogenesis on a cirrhotic background that could robustly discriminate cirrhosis from HCC. The expression of top modular genes was closely associated with HCC onset, development, and prognosis. Our work may provide a deeper understanding of molecular mechanisms in HCC onset from cirrhosis and offer new insights for screening and surveillance of high-risk patients with cirrhosis during anti-viral therapy.

## Data Availability

Publicly available datasets were analyzed in this study. This data can be found here: https://www.ncbi.nlm.nih.gov/geo/.

## Author Contributions

J-dJ conceived and designed the experiments. SS and WC performed data analysis and drafted the manuscript. All authors read and approved the final manuscript.

## Conflict of Interest Statement

The authors declare that the research was conducted in the absence of any commercial or financial relationships that could be construed as a potential conflict of interest.

## Footnotes

1 https://www.kegg.jp/

2 https://sourceforge.net/projects/mev-tm4/

3 https://cancergenome.nih.gov/

4 www.proteinatlas.org

## Supplementary Material

FIGURE S1 Liver carcinogenesis-related module identified from GSE89377, GSE17548, and GSE56140.

FIGURE S2 Liver carcinogenesis-related module identified from GSE98383, GSE17548, and GSE56140.

FIGURE S3 Liver carcinogenesis-related module identified from GSE98383, GSE89377, and GSE56140.

FIGURE S4 Verification of the identified modules for discriminating cirrhosis from HCC in GSE98383.

FIGURE S5 Verification of the identified modules for discriminating cirrhosis from HCC in GSE89377.

FIGURE S6 Verification of the identified modules for discriminating cirrhosis from HCC in GSE17548.

TABLE S1 Fold changes of the differentially expressed genes shared in GSE98383, GSE89377, and GSE17548.

TABLE S2 Significantly enriched KEGG pathways of the shared DEGs.

TABLE S3 Significantly enriched KEGG pathways of the genes in the identified gene module.

TABLE S4 Information of the genes in the identified module.

## Abbreviations

DEGs: differentially expressed genes
EMT: epithelial-to-mesenchymal transition
FC: fold-change
FDR: false discovery rate
HPA: Human Protein Atlas
GEO: Gene Expression Omnibus
HCC: hepatocellular carcinoma
KEGG: Kyoto Encyclopedia of Genes and Genomes
MAPK: mitogen-activated protein kinase
MCODE: molecular complex detection
PCA: principal component analysis
RMA: robust multichip average
STRING: Search Tool for the Retrieval of Interacting Genes
TCGA: the Cancer Genome Atlas
TPM: transcripts per million.
