# Supplementary figures and images for: Transcriptome Analysis Revealed a Highly Connected Gene Module Associated With Cirrhosis to Hepatocellular Carcinoma Development
Source: Front Genet. 2019 Apr 2;10:305. doi: 10.3389/fgene.2019.00305 (PMC6454075; doi:10.3389/fgene.2019.00305)

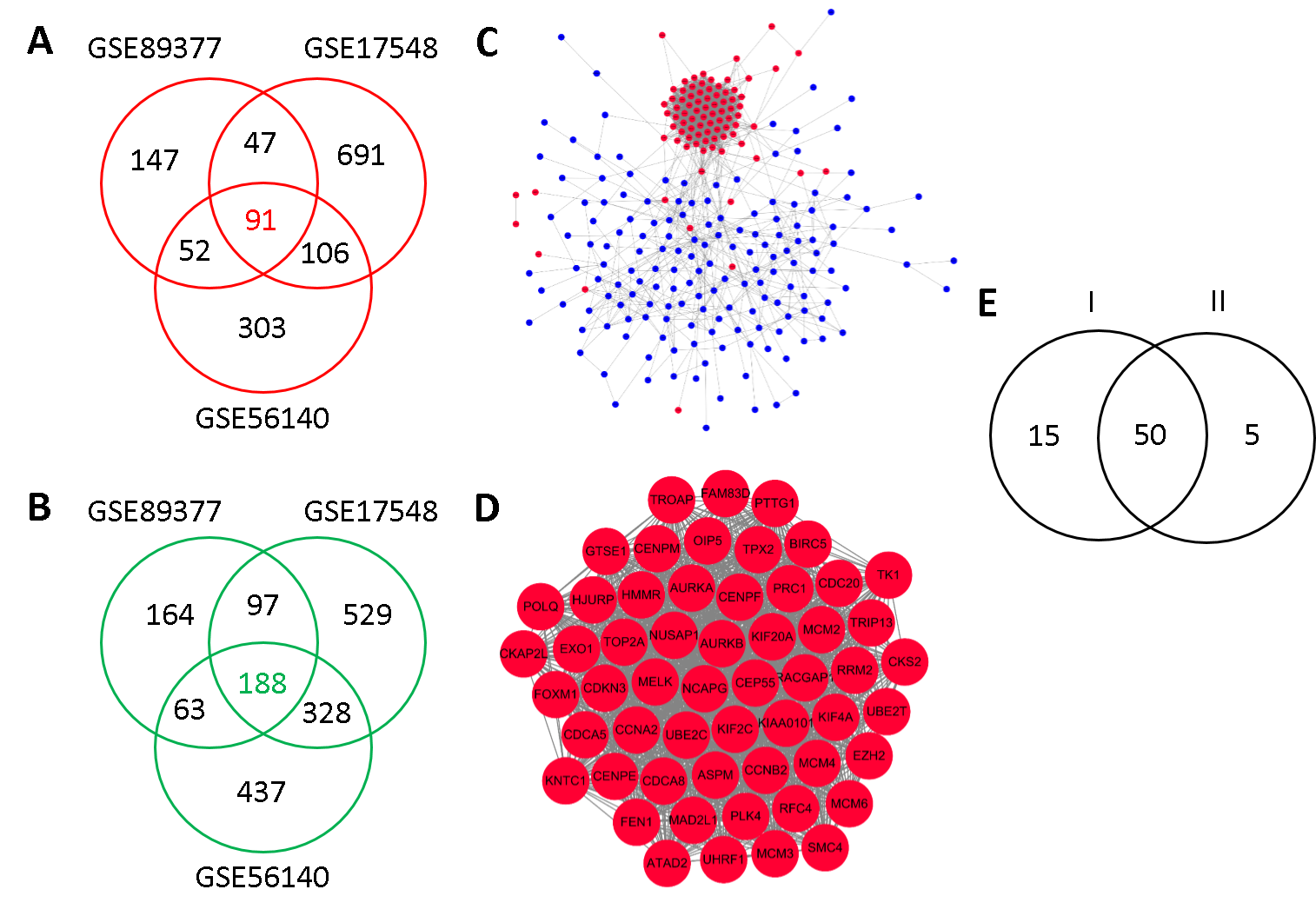

Supplement: FIGURE S1 — Liver carcinogenesis-related module identified from GSE89377, GSE17548, and GSE56140. [file Image_1.TIF]

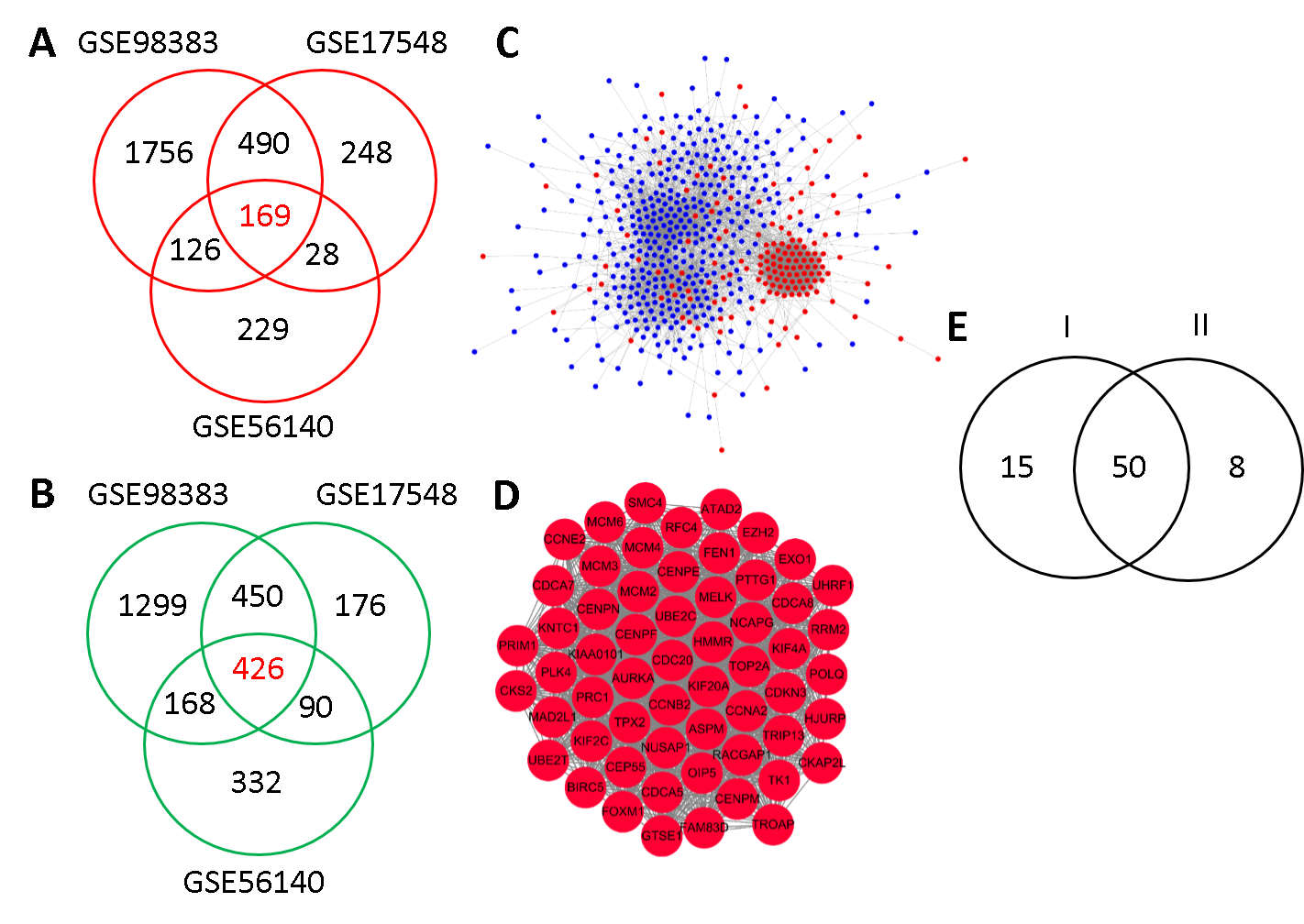

Supplement: FIGURE S2 — Liver carcinogenesis-related module identified from GSE98383, GSE17548, and GSE56140. [file Image_2.TIF]

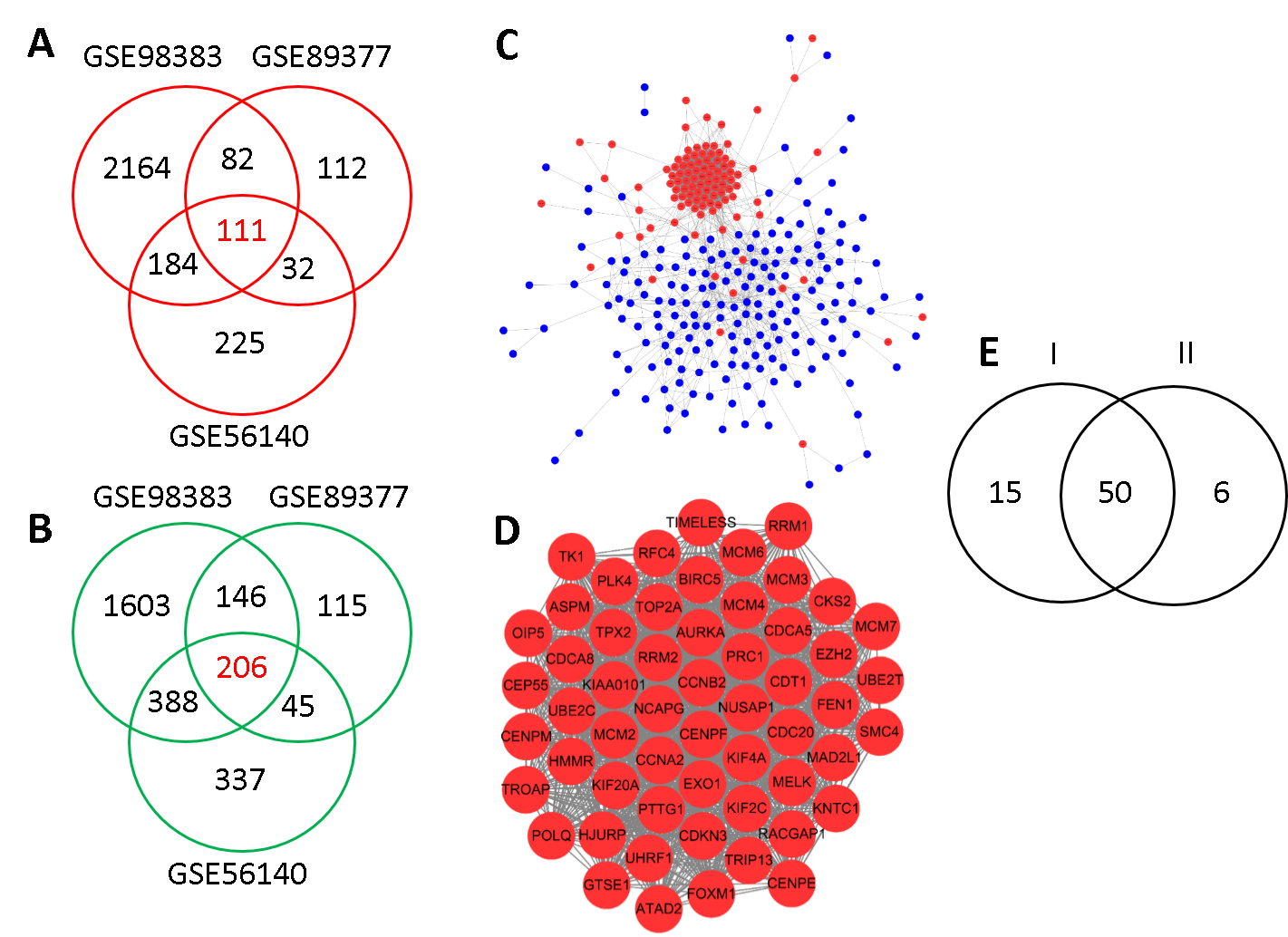

Supplement: FIGURE S3 — Liver carcinogenesis-related module identified from GSE98383, GSE89377, and GSE56140. [file Image_3.TIF]

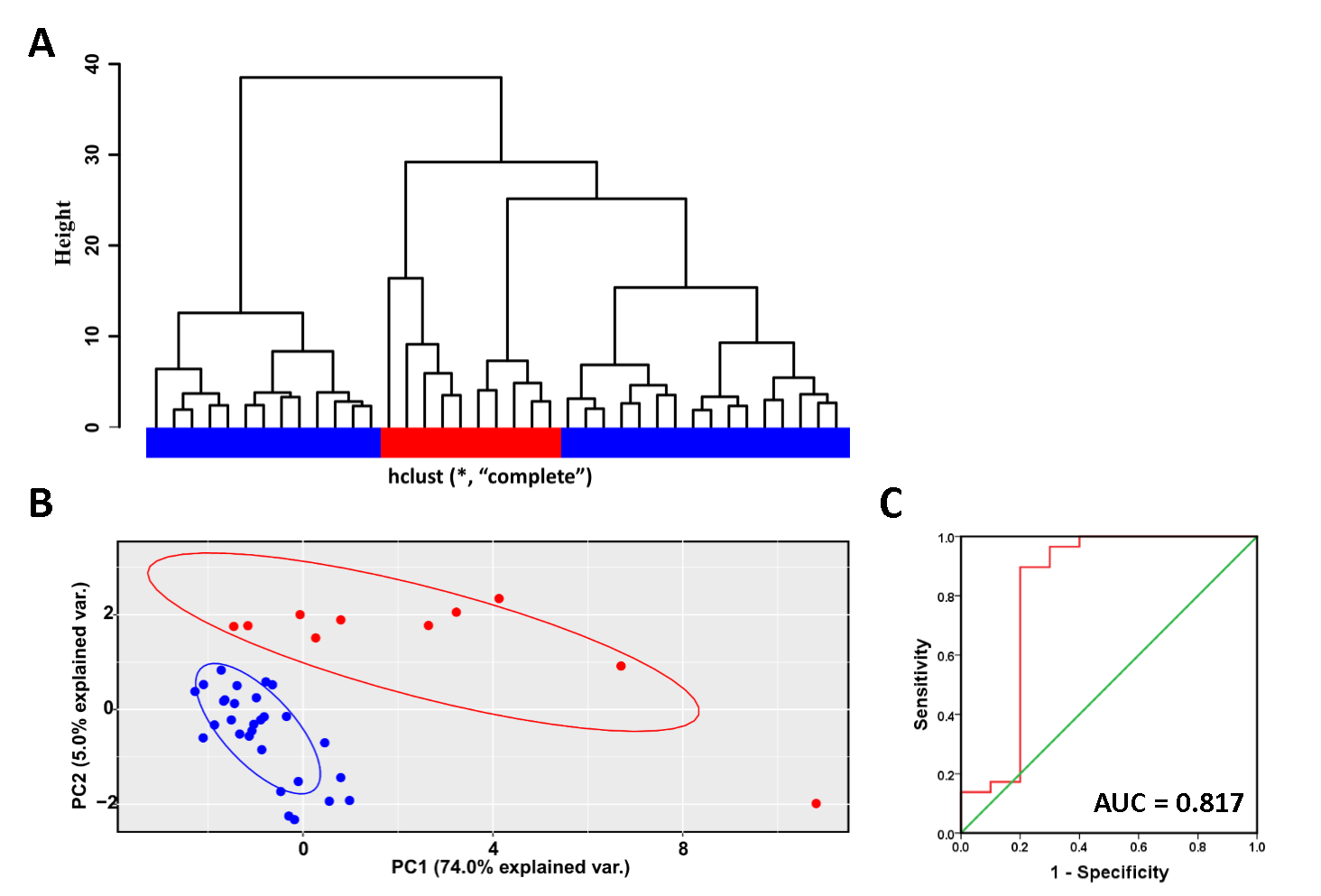

Supplement: FIGURE S4 — Verification of the identified modules for discriminating cirrhosis from HCC in GSE98383. [file Image_4.TIF]

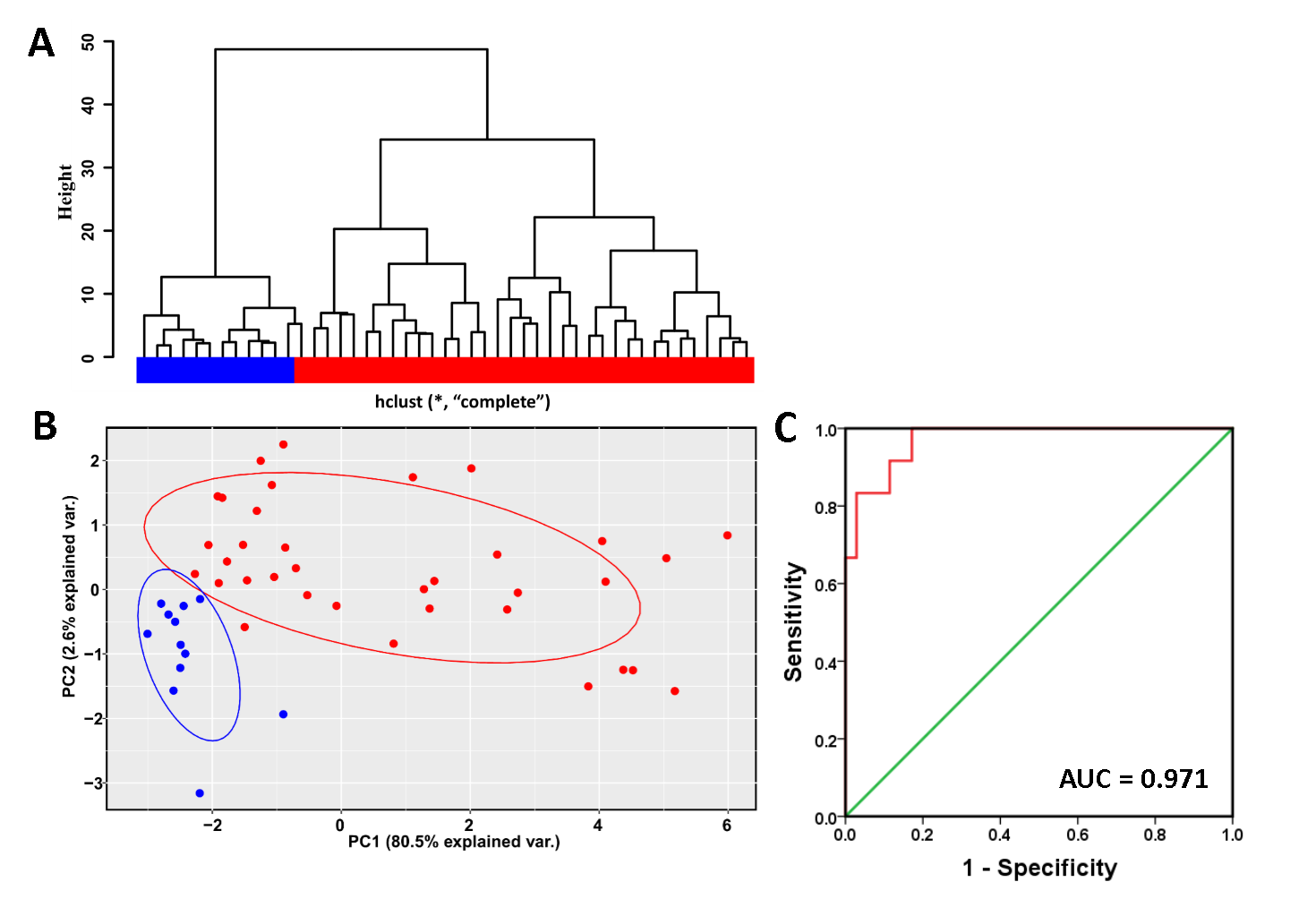

Supplement: FIGURE S5 — Verification of the identified modules for discriminating cirrhosis from HCC in GSE89377. [file Image_5.TIF]

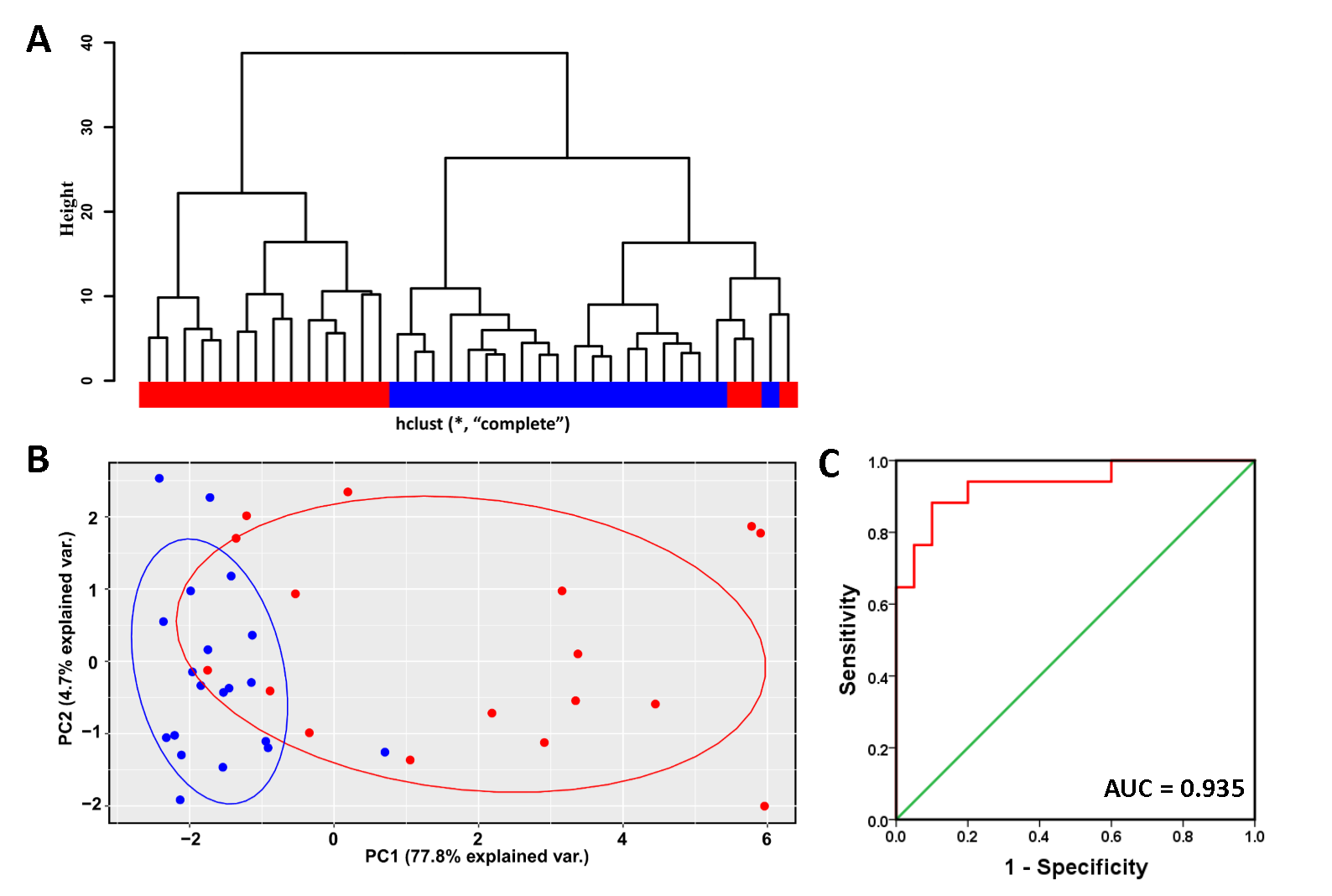

Supplement: FIGURE S6 — Verification of the identified modules for discriminating cirrhosis from HCC in GSE17548. [file Image_6.TIF]
